# Traumatic brain injury in the presence of Aβ pathology affects neuronal survival, glial activation and autophagy

**DOI:** 10.1038/s41598-021-02371-3

**Published:** 2021-11-26

**Authors:** Linn Streubel-Gallasch, Marlena Zyśk, Chiara Beretta, Anna Erlandsson

**Affiliations:** Department of Public Health and Caring Sciences/Molecular Geriatrics, Rudbeck Laboratory, Uppsala University, 751 85 Uppsala, Sweden

**Keywords:** Autophagy, Astrocyte, Alzheimer's disease, Neurodegeneration, Biological models, Brain injuries

## Abstract

Traumatic brain injury (TBI) presents a widespread health problem in the elderly population. In addition to the acute injury, epidemiological studies have observed an increased probability and earlier onset of dementias in the elderly following TBI. However, the underlying mechanisms of the connection between TBI and Alzheimer’s disease in the aged brain and potential exacerbating factors is still evolving. The aim of this study was to investigate cellular injury-induced processes in the presence of amyloid β (Aβ) pathology. For this purpose, a co-culture system of cortical stem-cell derived astrocytes, neurons and oligodendrocytes were exposed to Aβ_42_ protofibrils prior to a mechanically induced scratch injury. Cellular responses, including neurodegeneration, glial activation and autophagy was assessed by immunoblotting, immunocytochemistry, ELISA and transmission electron microscopy. Our results demonstrate that the combined burden of Aβ exposure and experimental TBI causes a decline in the number of neurons, the differential expression of the key astrocytic markers glial fibrillary acidic protein and S100 calcium-binding protein beta, mitochondrial alterations and prevents the upregulation of autophagy. Our study provides valuable information about the impact of TBI sustained in the presence of Aβ deposits and helps to advance the understanding of geriatric TBI on the cellular level.

## Introduction

Alzheimer’s disease (AD) is the most common form of dementia, affecting 40–50 million people globally^1,2^. Epidemiological studies have long pointed to a correlation between traumatic brain injuries (TBI) sustained in early adulthood and the long-term risk of dementia^3–5^. However, whether TBI sustained later in life (geriatric TBI) also augments the probability of developing dementia has been studied less extensively. The prevalence of TBI is highest in the younger population (till 25 years of age) and in the elderly (> 70 years)^6–8^. Incidences of TBI in older age groups are associated with worse clinical outcomes, but only a few studies have investigated the likelihood of dementia following geriatric TBI^9,10^. These studies have recorded an increased risk and earlier onset of dementias in TBI patients, but knowledge about the underlying mechanisms and potential exacerbating factors is still evolving. Several characteristics of the aging brain may influence the response to TBI, such as increased inflammation and oxidative stress, genome instability and the accumulation of cellular waste products due to ineffective lysosomal degradation^11^. Especially, the higher load of amyloid beta (Aβ) in the aging brain has received much attention. Albeit a hallmark of patients with AD, Aβ deposits have also frequently been observed in neurologically healthy aged individuals^12–14^. In order to identify specific cellular and molecular processes that could link TBI to AD development, in vitro disease models are very useful. In this study, we investigate the consequences of TBI in the presence of Aβ deposits, using a neuro-glial cell culture system. Our study thus mimics the situation in the aging brain and enables the investigation of central cellular pathways. Using the same co-culture system, we have previously shown that astrocytes rapidly engulf large amounts of Aβ_42_ protofibrils, followed by storage rather than degradation of the ingested material^15–18^. Since inefficient lysosomal degradation has also been reported in various neurodegenerative diseases including AD, we put a special focus on the investigation of autophagic activity in this study.

The term autophagy describes an evolutionary conserved process used by cells to degrade cytoplasmic material, aberrant proteins and organelles such as damaged mitochondria within the lysosome^19,20^. Autophagy plays a fundamental role in maintaining homeostasis and thus normal cellular function. Autophagic activity decreases with age and the progressive reduction may well contribute to the observed accumulation of proteins in the aging brain^21,22^. In relation to TBI, both beneficial and damaging roles for autophagy have been described. Autophagy being upregulated at the site of injury might provide ways to remove damaged cells and harmful debris, but on the other hand, autophagosome accumulation and thus decreased autophagic flux have also been reported following injury^23,24^. In conclusion, the role of autophagy in neurodegenerative diseases and following TBI is of a complex nature, seemingly beneficial at early stages, but detrimental at later stages of the diseases. This study provide valuable information about the impact of TBI sustained in the presence of Aβ deposits and may help to advance our understanding of geriatric TBI.

## Results

### An in vitro model to study the effects of TBI in the presence of Aβ pathology

**Figure 1:**
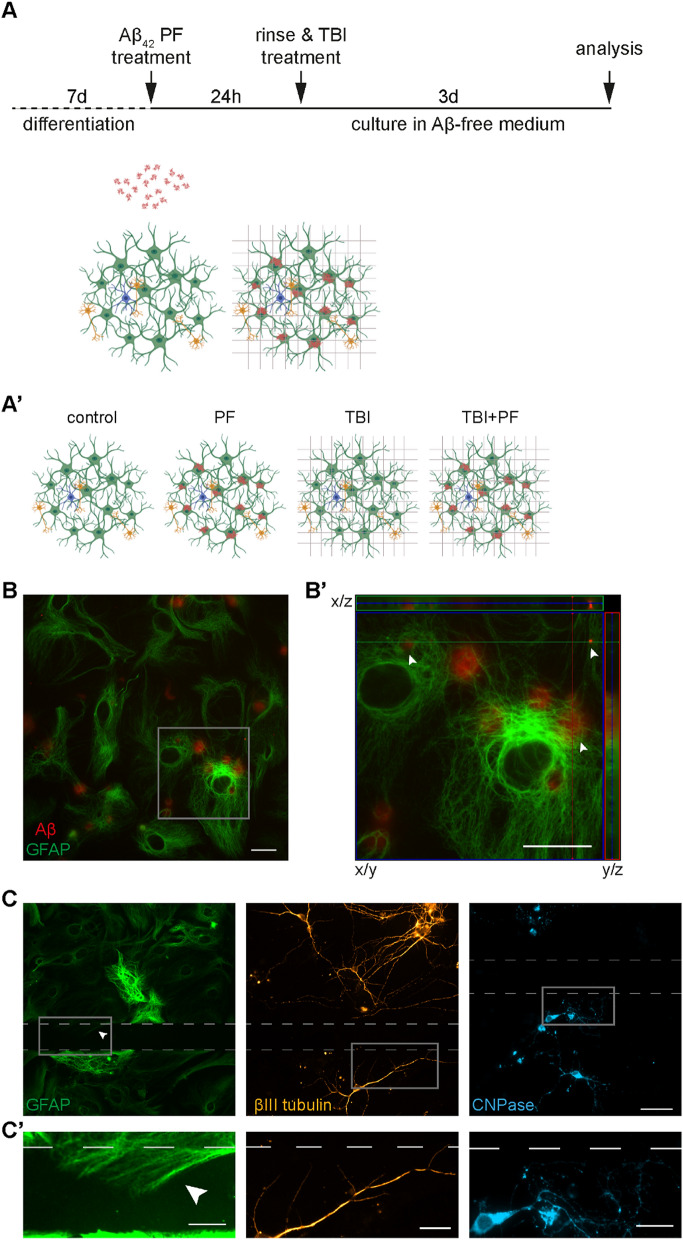
An in vitro model to study the effects of TBI in the presence of Aβ pathology. (**A**) Schematic outline of the experimental set-up. Differentiated cell cultures (astrocytes (green), neurons (gold) and oligodendrocytes (cyan); represented according to observation) were exposed to 0.1 µM Aβ_42_ protofibrils for 24 h. The cells were then rinsed extensively before being subjected to experimental TBI, a mechanically induced scratch injury performed with a scalpel. Subsequently, the cells were cultured for three additional days. (**A′**) Depiction of the four groups used in this study: untreated (control), scratch injured (TBI), exposed to Aβ_42_ protofibrils (PF) and the combination of both (TBI + PF). (**B**) Representative image of the co-culture exposed to Aβ_42_ protofibrils and stained for astrocytes (GFAP) and Aβ. (**B′**) A higher magnification of the area outlined by the square in (**B**), showing a close-up of the Aβ deposits (some of different sizes are pointed out by the arrow heads). Orthogonal projections were made along the lines depicted in the main image and show the Aβ inclusion surrounded by GFAP. (**C**) Representative images of astrocytes (GFAP), neurons (βIII tubulin) and oligodendrocytes (CNPase). The scalpel cuts left noticeable injuries, which are outlined by the dashed lines. The three cell types react differently to the injury. Astrocytes extend towards and along the cut and the arrow head draws attention to an astrocyte growing into the area of laceration. Neurons also grow towards and along, but do not breach, the site of injury. Oligodendrocytes avert the area of the cut, unless in its direct vicinity, but were not observed to grow across the injury site. (**C**′) Magnification of the areas outlined in (**C**). Scale bars 20 µm (**B**,**B**′,**C**′) and 50 µm (**C**).

Combining our well-characterized in vitro models of TBI and Aβ pathology^15–18,25,26^ allowed us to study the consequences of physical injury in the presence of Aβ deposits. The experimental set-up is schematically depicted in Fig. 1A. In short, co-cultures of astrocytes, neurons and oligodendrocytes were exposed to 0.1 µM Aβ_42_ protofibrils for 24 h. After the Aβ exposure pulse, the cultures were extensively washed and subjected to a standardized scratch injury using a scalpel. The four experimental groups compared in this study were untreated (control), scratch injury (TBI), Aβ_42_ protofibril exposure (PF) and the combination of both (TBI + PF) (Fig. 1A′).

Analogous to our earlier studies^15–18^, astrocytes engulfed and accumulated great amounts of Aβ (Fig. 1B). In contrast, Aβ deposits in neurons were undetectable, while the small number of oligodendrocytes in the culture also accumulated Aβ to some degree^16,17^. Intracellular Aβ inclusions in astrocytes were surrounded by glial fibrillary acidic protein (GFAP)-positive intermediate filaments as verified by z-stack imaging (Fig. 1B′). Of note, GFAP, which is part of the cytoskeleton, only covers a portion of the astrocyte, but does not depict the cell’s perimeter^27^. Astrocytes contained mostly large, cottony deposits, but some medium-sized and small inclusions were also observed (Fig. 1B′, arrow heads), which was in line with previous reports^17^.

Astrocytes, neurons and oligodendrocytes reacted differently to the scratch injury (Fig. 1C,C′; the sites of injury are marked by the dashed lines). Astrocytes and neurons were clearly drawn towards the area of laceration, while oligodendrocytes, unless in its direct proximity, were avoiding it. However, neurons were only ever found to grow along the site of injury, whereas astrocytes were often observed to breach the cut (Fig. 1C′).

### Reduced number of neurons in cultures exposed to both Aβ_42_ protofibrils and TBI

**Figure 2:**
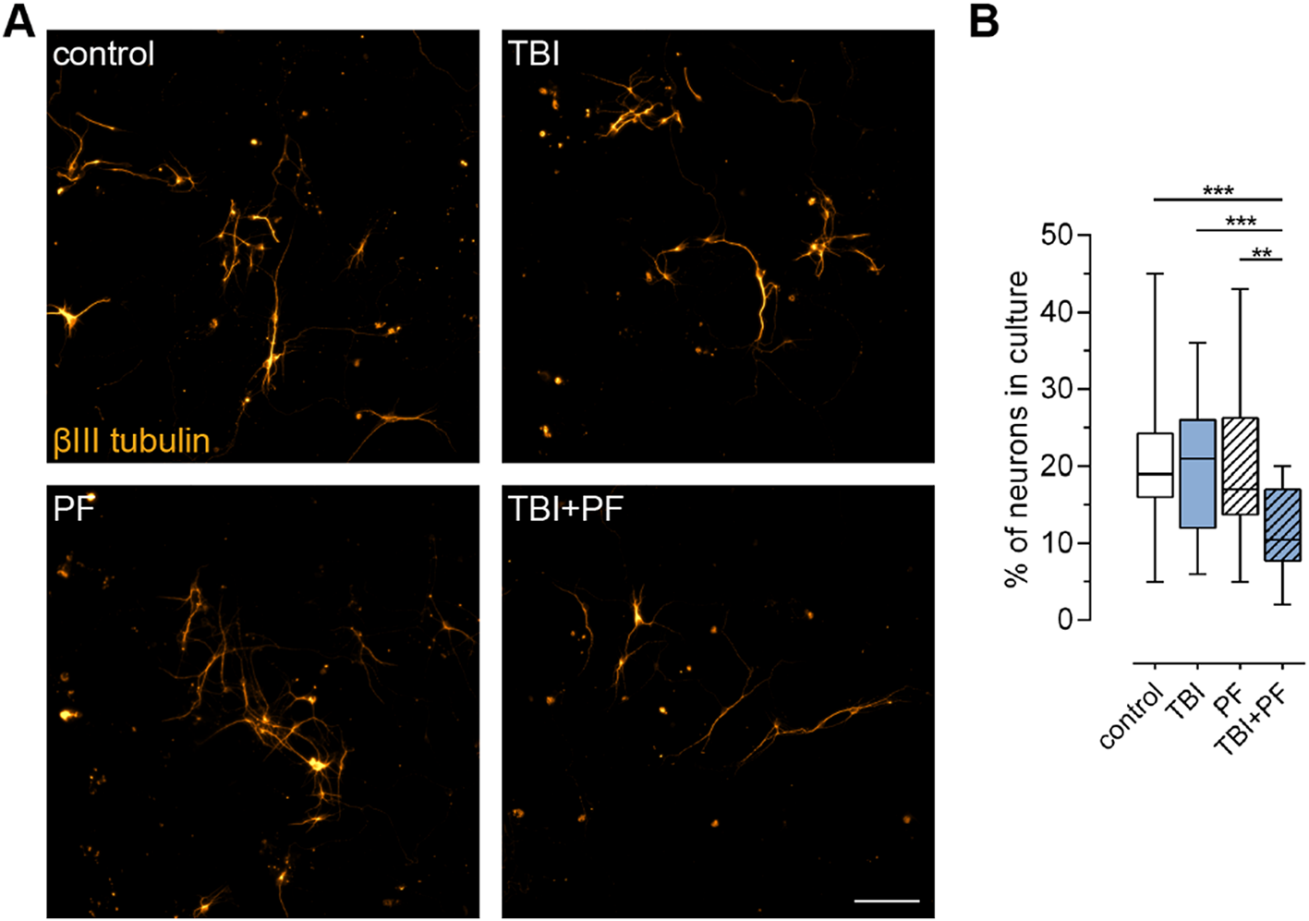
The number of neurons in culture declined following the combined exposure to Aβ_42_ protofibrils and TBI. (**A**) Representative fluorescence microscopy images of neurons (βIII tubulin) and (**B**) the percentage of neurons in culture (calculated by reference to the total number of viable cells). The percentage of neurons in cultures exposed to Aβ_42_ protofibrils and TBI alone did not differ from control cultures, while the combination (TBI + PF) resulted in a lower percentage of neurons. Ten images per independent cell culture (n = 3) and experimental group were analyzed and reported. Statistical analysis was performed using the Kruskal–Wallis test followed by Dunn’s multiple comparisons test, *p ≤ 0.05 **p ≤ 0.01 ***p ≤ 0.001. Scale bar 100 µm.

We have previously shown that the viability of neurons remains relatively unchanged in this in vitro trauma model system and that astrocytes have a protective effect on the neuronal survival by engulfing great amounts of cell corpses and thereby reducing the chance of bystander killing^25^. Furthermore, the exposure to Aβ_42_ protofibrils does not induce direct neuronal cell death in the co-cultures. However, a few weeks later the incomplete degradation of Aβ_42_ protofibrils by astrocytes results in the release of extracellular vesicles containing neurotoxic Aβ^15,17^. Here, we studied the effect of TBI on neuronal survival in Aβ_42_ protofibril-exposed cultures by immunocytochemistry using the neuron specific marker βIII tubulin. The number of βIII tubulin-positive neurons were counted and compared between the four experimental groups (Fig. 2A). Interestingly, we identified a statistically significant lower percentage of neurons in cultures exposed to Aβ_42_ protofibrils and injury in combination (TBI + PF), but not in control, TBI or PF cultures (Fig. 2B; Kruskal–Wallis test p < 0.001).

### Differential expression of the astrocytic markers GFAP and S100β following Aβ_42_ protofibril and TBI exposure

**Figure 3:**
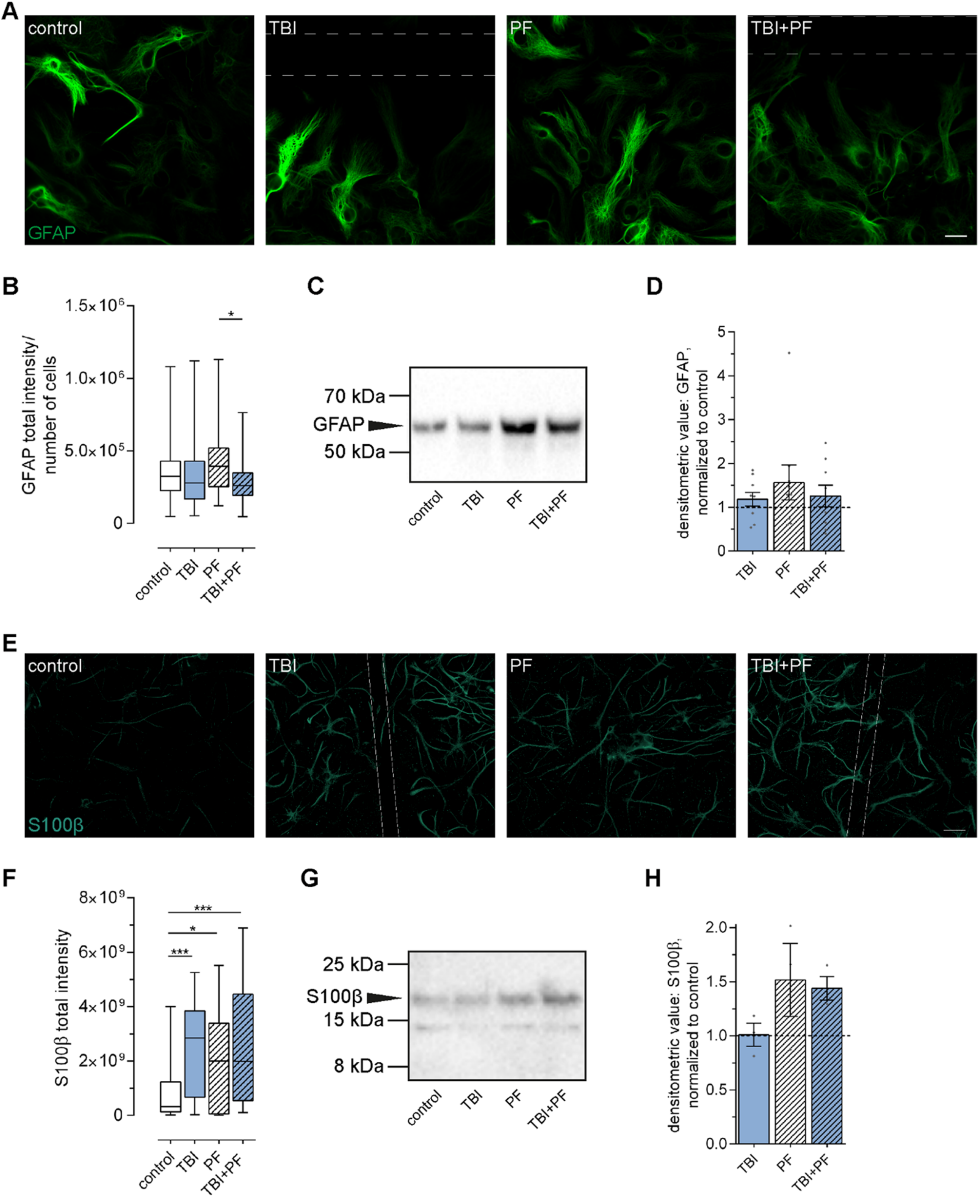
Differential expression of the astrocytic markers GFAP and S100β following Aβ_42_ protofibril and TBI exposure. (**A**) Representative images of the astrocytes (GFAP) in culture showing a wide range of GFAP expression across all experimental groups. The sites of the mechanically induced scratch injury are indicated by the dashed lines. (**B**) The corresponding quantification determined a trend towards higher GFAP fluorescence intensity in Aβ_42_ protofibril-exposed astrocytes, with a statistically significant difference compared to the double exposure (TBI + PF). Ten images per independent cell culture (n = 3) and experimental group were analyzed and reported. (**C**,**D**) Western blot analysis also pointed towards an increase in GFAP expression in the PF group (the control group is represented by the dashed line at 1); mean ± SEM (independent cell cultures, n = 9). (**E**) Representative images of cell cultures stained for the astrocytic marker S100β and (**F**) the quantification of S100β fluorescence intensity. All exposures caused an upregulation of S100β expression. Ten images per independent cell culture (n = 4) and experimental group were analyzed and reported. (**G**,**H**) The follow-up western blot analysis also pointed towards an increase in S100β expression for the PF and TBI + PF groups compared to control (represented by the line at 1.0); mean ± SEM (independent cell cultures, n = 3). Statistical analyses of GFAP and S100β fluorescence intensities and GFAP Western blot data were performed using the Kruskal–Wallis test followed by Dunn’s multiple comparisons test. Western blot of S100β expression was analyzed using one-way ANOVA followed by Tukey’s multiple comparison test, *p ≤ 0.05 **p ≤ 0.01 ***p ≤ 0.001. Scale bars 20 µm (**A**) and 30 µm (**E**).

Next, we examined how the combined exposure to Aβ_42_ protofibrils and TBI affect astrocytes. Astrocytes are fundamental to maintaining homeostasis and damaged or overwhelmed astrocytes could be the cause for atrophy and neurodegeneration. Astrocytes are known to possess a high degree of plasticity and adjust their characteristics in order to maintain their functions in the brain and preserve homeostasis as long as possible^28^. We thus went on to study the expression of the well-recognized astrocytic markers GFAP and S100β^29^. Immunocytochemistry established that GFAP expression of the astrocytes varied over a wide range in all experimental groups (Fig. 3A). While the GFAP intensity of some astrocytes was very strong, others possessed only low levels. Higher GFAP expression has often been associated with reactive astrocytes^30–32^. However, we could not establish that astrocytes upregulate their GFAP expression specifically when in direct vicinity to the scratch site or possessing high Aβ loads. The observed upregulation in certain cells is thus more likely caused by more general changes of the different cultures. As a consequence, the overall difference between the experimental groups was quantified by assessing total GFAP intensity and a statistically significant difference was determined (Fig. 3B; Kruskal–Wallis test p = 0.0328). Dunn’s multiple comparisons test identified a decrease in GFAP expression in in TBI + PF cultures compared to PF cultures, but did not reveal differences between any of the other experimental groups. Western blot analysis of GFAP in cell lysates also pointed towards an upregulation upon Aβ protofibril exposure compared to the other experimental groups, albeit not statistically significant (Fig. 3C,D; Kruskal–Wallis test p = 0.6967).

The calcium-binding protein S100β is another commonly used marker of astrocytes and reports have associated upregulated S100β levels with astrogliosis and with pathophysiological conditions of the brain in general^33–35^. In this study we evaluated S100β levels by immunocytochemistry and western blot analysis. Representative images depicting the staining for S100β (Fig. 3E) and the corresponding quantification of total S100β fluorescence intensity (Fig. 3F) identified an upregulation of S100β levels in all experimental groups compared to control cultures (Kruskal–Wallis test p < 0.001). Successive western blot analysis of S100β in cell lysates (Fig. 3G) also pointed towards an upregulation in the PF and TBI-PF groups, however not statistically significant (Fig. 3H; one-way ANOVA p = 0.1660).

These observations are an indication of how astrocytes, especially following the combined exposure to Aβ_42_ protofibrils and TBI, are reacting to changes in their environment. It needs to be further determined, whether these exposures direct astrocytes into a specific GFAP_low_/S100β_high_ reactivity state or if the diverse expression of GFAP and S100β reflects the health status of astrocytes following the combined exposure.

### Increased GFAP levels in the cell culture medium and extracellular vesicles following TBI

**Figure 4:**
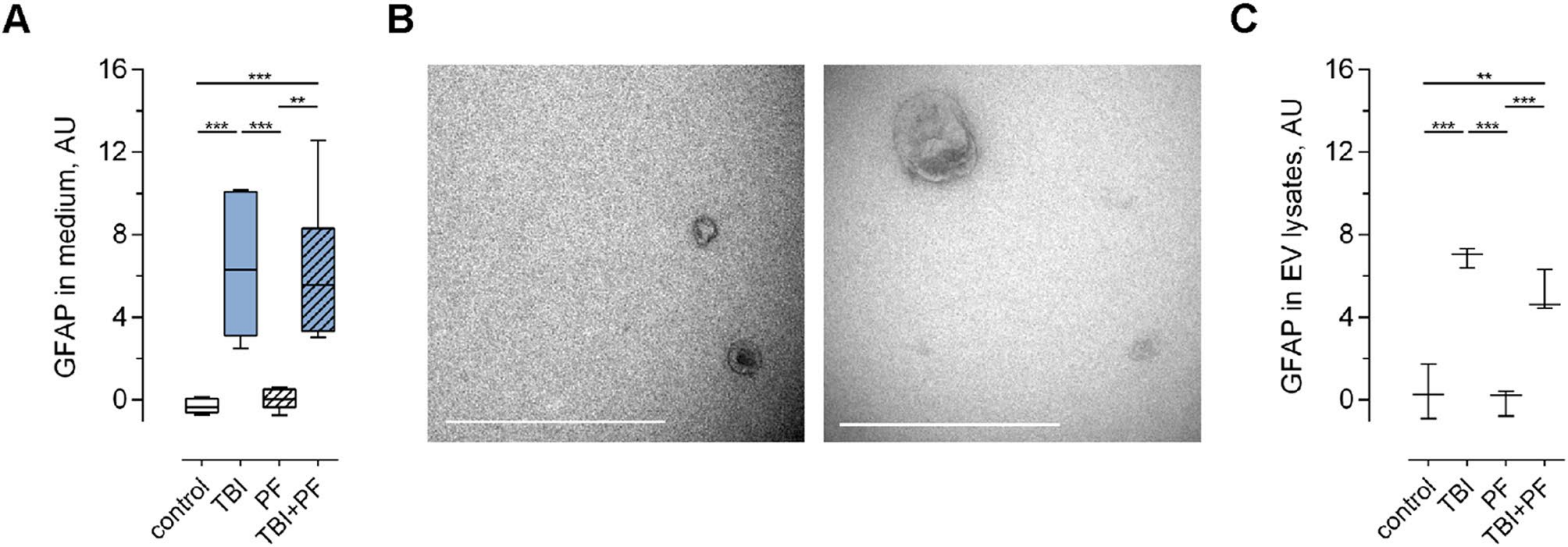
Increased levels of the astrocytic marker GFAP in the cell culture medium and in extracellular vesicles following TBI. (**A**) The level of GFAP in the cell culture medium was assessed by ELISA and was found to be increased following TBI exposure, but not in the control or PF experimental groups (independent cell cultures, n = 6). (**B**) Cell culture medium was ultracentrifuged and the successful isolation of EVs of various sizes was verified by TEM. (**C**) Following isolation, EVs were lysed and GFAP levels were analysed using ELISA (independent cell cultures, n = 3). Quantification established increased levels in the TBI and TBI + PF experimental groups compared to control and Aβ protofibril exposure. Statistical analyses was performed using one-way ANOVA followed by Tukey’s multiple comparison test, *p ≤ 0.05 **p ≤ 0.01 ***p ≤ 0.001. Scale bars 500 nm (**B**).

In order to evaluate if astrocytes, in response to Aβ protofibril exposure or trauma, release GFAP into the cell culture medium, either free-floating or in extracellular vesicles (EVs), we analysed medium and EV lysate samples by enzyme-linked immunosorbent assay (ELISA). We found GFAP levels to be increased in the medium of cell cultures subjected to TBI, but not in control and cells exposed to Aβ_42_ protofibrils alone (Fig. 4A; one-way ANOVA p < 0.001). EVs isolated from the conditioned media (Fig. 4B) were lysed and also analysed by ELISA and higher levels of GFAP were also found in EVs released from cells that had undergone TBI (Fig. 4C; one-way ANOVA p < 0.001). Our data show that TBI, but not Aβ exposure, induces the secretion of GFAP. This could be due to either active secretion (in response to the injury) or passive secretion (because of damaged cell membranes) or both.

### Aβ_42_ protofibril exposure and TBI cause mitochondrial defects

**Figure 5:**
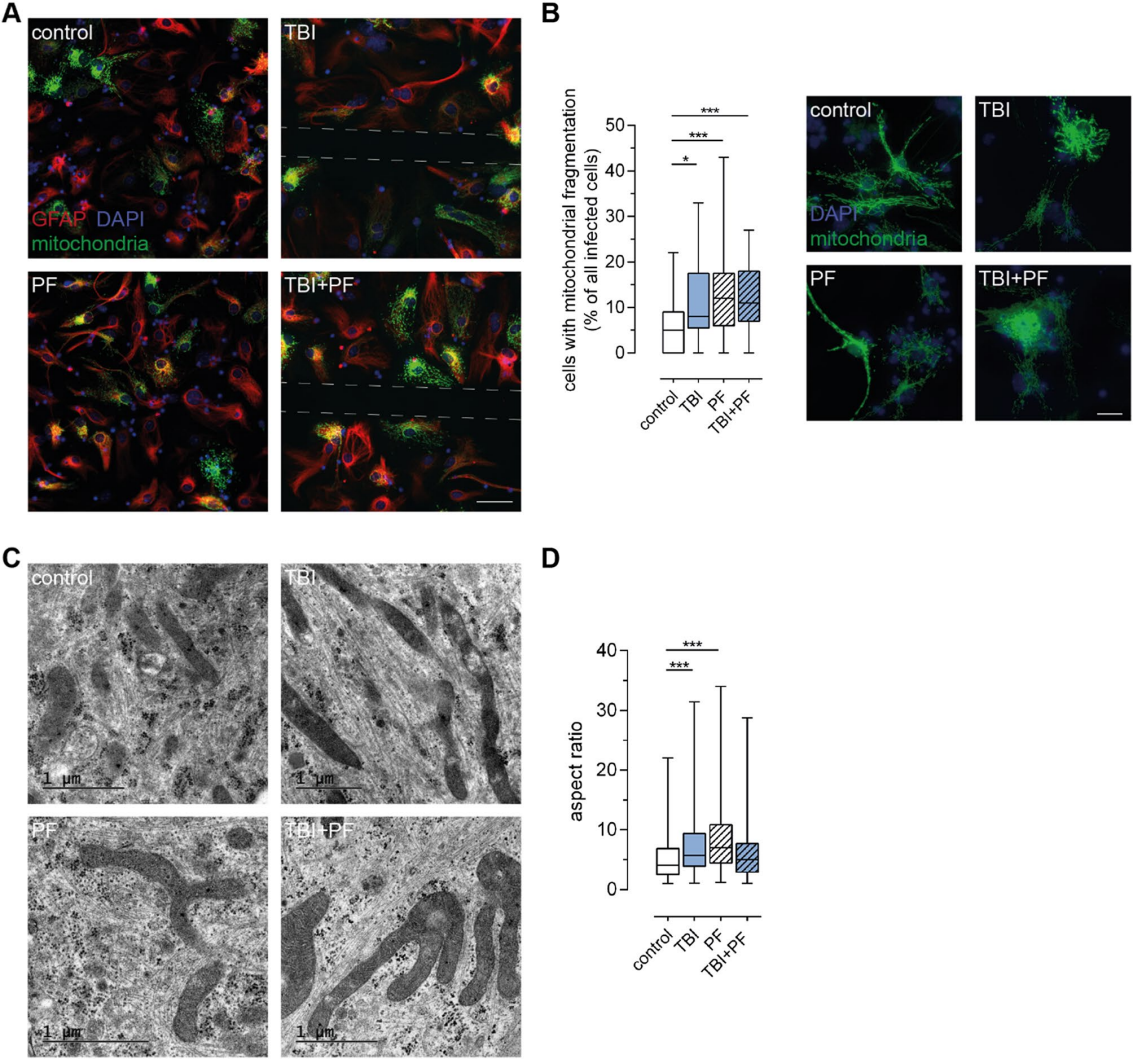
TBI and Aβ_42_ protofibril exposure cause mitochondrial damage in astrocytes. (**A**) The health of mitochondria in astrocytes (GFAP) in response to the TBI and Aβ_42_ protofibril exposure was investigated. Representative images show mitochondria labeled with CellLight Mitochondria-GFP and cell nuclei with DAPI. The sites of the injury are indicated by the dashed lines. (**B**) Mitochondrial network fragmentation was assessed and quantified. While control cells possess a predominately healthy-looking mitochondrial network with strands of branching mitochondria, exposed cells display various states of mitochondrial network disruption. Increasing fragmentation can be observed and at late stages defective mitochondrial membranes causes the release of labeled mitochondrial proteins that become visible in the cytosol. Fifteen images per independent cell culture (n = 3) and experimental group were analyzed and reported. (**C**) Representative TEM images showing examples of mitochondria observed in control cells and cells undergone TBI, Aβ_42_ protofibril exposure or the combination of both. Abnormal fusion events were primarily observed in exposed cells and some mitochondria possessed marked lesions. (**D**) Quantification of the aspect ratio (mitochondrial length/width) confirmed these observations. Five cells per experimental group were analyzed; quantifying the mitochondria in a total of 55–68 TEM images per group. Statistical analyses were performed with the Kruskal–Wallis test followed by Dunn’s multiple comparisons test, *p ≤ 0.05 **p ≤ 0.01 ***p ≤ 0.001. Scale bars 50 µm (**A**) and 20 µm (**B**).

One way to assess changes to cellular homeostasis is to examine the mitochondrial network. A disrupted mitochondrial network is a sign of cellular stress and imbalance between mitochondrial fission and fusion^36^. Hence, mitochondria were labeled by transduction with the baculovirus-based CellLight Mitochondria-GFP reagent and the state of the mitochondrial network was assessed in control, TBI, PF and TBI + PF cultures. Representative images of astrocytes with labeled mitochondria are shown in Fig. 5A. Intact mitochondria exhibit a long thread-like morphology and form an interconnected web with extensive branching, while network disruption is characterized by increasing mitochondrial fragmentation, recognized as unconnected dot or rod-like structures as well as clump formation^37^. The number of cells possessing damaged mitochondria was significantly increased following Aβ_42_ protofibril, TBI as well as the combined exposure (Fig. 5B; Kruskal–Wallis test p < 0.001). However, there was no significant difference in mitochondrial network impairment between the three groups. We next sought to investigate the mitochondria by transmission electron microscopy (TEM). Confirming the immunocytochemistry results, morphologically altered mitochondria were primarily observed in cells that had undergone Aβ_42_ protofibril exposure and TBI alone or in combination (Fig. 5C). In those exposed cells, odd shaped mitochondria were commonly observed, probably due to abnormal fusion events, and in some cases the mitochondria possessed marked lesions. This altered mitochondrial morphology was confirmed by quantification of the mitochondrial aspect ratio (mitochondrial length/width) (Fig. 5D; Kruskal–Wallis test p < 0.001). Taken together, our data demonstrate severe mitochondrial alterations following physical injury or Aβ exposure, but no clear additive effect of the two. The observed changes, such as the clearly affected fission/fusion homeostasis and impaired lysosomal clearance of damaged mitochondria, reflect that the astrocytes are under severe cellular stress. Neurodegenerative diseases, including AD, are defined by loss of brain homeostasis, which could at least partly be due to the severely stressed astrocytes unable to fulfil their normal tasks.

### Upregulated autophagy upon Aβ_42_ protofibril exposure or TBI, but not following the combined exposure

Autophagy, the conserved pathway for the elimination of aberrant proteins and organelles, such as damaged mitochondria, has been linked to neurodegenerative diseases and TBI^24,38–40^. Hence, we next investigated autophagy in our model system. When studying autophagic activity, the analysis of the autophagy-related proteins microtubule-associated protein light chain 3 (LC3B) and polyubiquitin-binding protein (p62) plays a pivotal role^41–44^. Induction of autophagy causes the conjugation of LC3B I to a phosphatidylethanolamine moiety to form LC3B II, which, now being lipophilic, is incorporated into the membrane of the forming autophagosome^45^. One way to sequester damaged cytosolic material as well as aggregated proteins for autophagy is by marking them for degradation via ubiquitination^46^. As one of the first cargo adaptor proteins described, p62 is recruited to the ubiquitinated material and subsequently, through its LC3-recognition sequence, shuttles the material to the autophagosome for degradation through the autophagy-lysosomal pathway^47–50^. LC3B and p62, together with the associated cargo, are being degraded during autophagy and can thus be used as reporters of degradation capacity.

**Figure 6:**
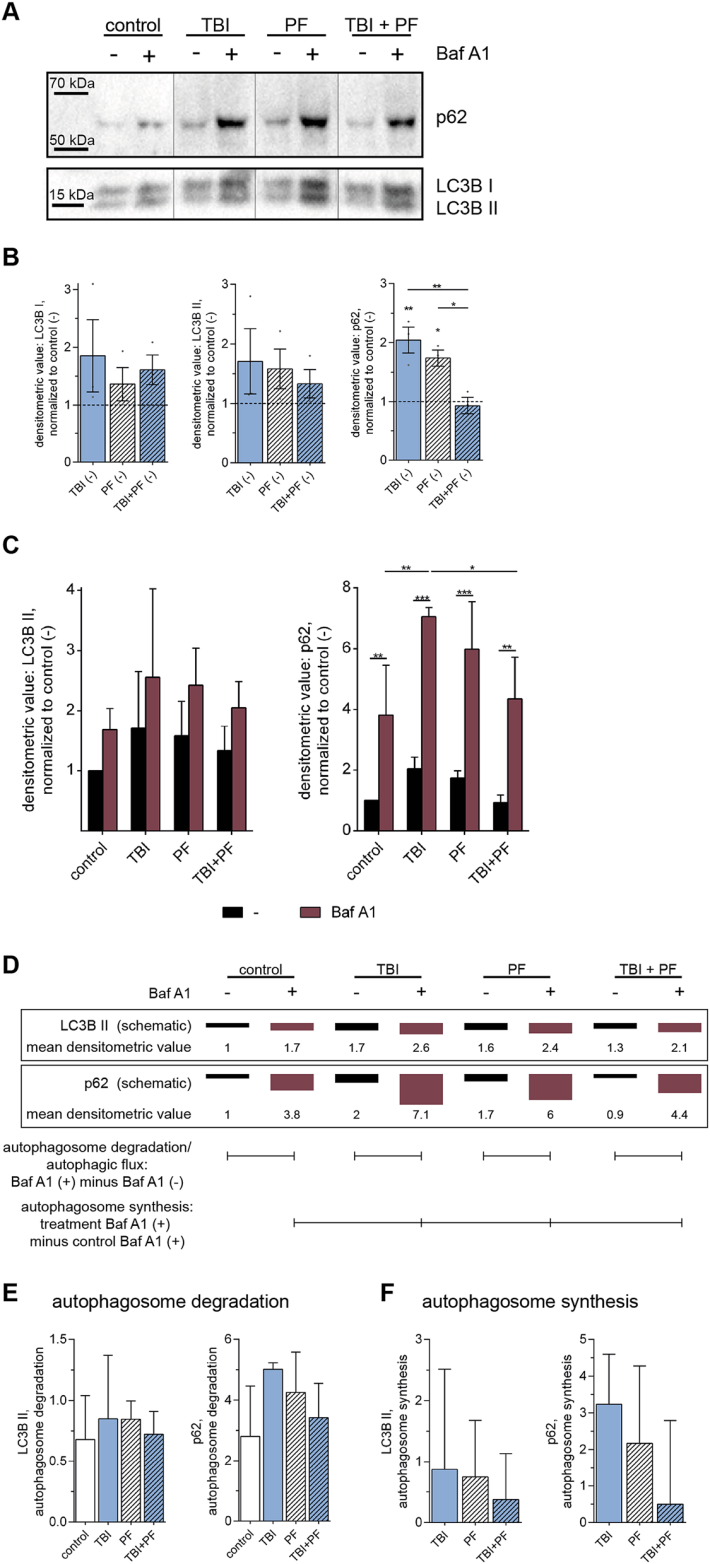
Upregulated autophagic activity in the TBI and PF groups, but not in TBI + PF. (**A**) The autophagy-related proteins p62 and LC3B II can be used as reporters to measure autophagic activity by immunoblotting. Usage of the autophagy inhibitor Baf A1 allows the evaluation of autophagic flux. Experiments were performed on cell lysates of three independent cell cultures and representative blots are shown. (**B**) LC3B I, LC3B II and p62 bands detected on the immunoblots were quantified from three independent experiments and the obtained densitometric values are displayed as mean ± SEM. (**C**) Static observations of the cellular levels of LC3B and p62 cannot be used to draw conclusions about the actual lysosomal turn-over. However, when comparing the results in the absence (−) and presence of the inhibitor Baf A1 we determined an increase in autophagic flux. (**D)** Schematic representation of the data shown in (**C**). Mean values are presented and were used to illustrate how autophagosome synthesis and degradation can be calculated. (**E**) The amount of autophagosomes that would have been degraded shows a tendency for an increase in the TBI and PF groups, but not in TBI + PF. (**F**) Correspondingly, autophagosome synthesis calculations showed the same trend, though also not statistically significant. Data in (**C**), (**E**) and (**F**) are displayed as mean with SD and were obtained from three independent cell cultures. Statistical analysis was performed using one-way ANOVA (**B**,**E**,**F**) and two-way ANOVA (**C**) followed by Tukey’s multiple comparison tests, *p ≤ 0.05 **p ≤ 0.01 ***p ≤ 0.001.

To receive a full picture of the autophagic activity, we choose to measure LC3B and p62 as well as employing the autophagy inhibitor bafilomycin (Baf A1) (Fig. 6A). Albeit its larger molecular mass LC3B I runs at 16 kDa, while LC3B II runs at 14 kDa, which is probably due to its hydrophobic nature^42^. The graphs represent the measured densitometric values of the bands detected through immunoblotting. Protein normalization to GAPDH, one of the standard house-keeping proteins, was found to be unsuitable in this study, since both, the experimental conditions (TBI, PF and TBI + PF) and the use of the autophagy inhibitor Baf A1, were found to influence the expression level of the GAPDH (Supplementary Fig. 2A,B). Equal loading using the protein concentrations determined by BCA Protein Assay was confirmed by total protein stain (Supplementary Fig. 2C,D). First, we evaluated the levels of LC3B I, LC3B II and p62 in the four groups (control, TBI, PF and TBI + PF) at basal conditions (−) without the addition of the autophagy inhibitor (Fig. 6B). We identified an increase of LC3B I and LC3B II for TBI, PF and TBI + PF compared to control cultures, although not statistically significant (one-way ANOVA p = 0.4488 and p = 0.5115, respectively). Often the LC3B II/I ratio is being reported, which uses the conversion of LC3B I to LC3B II as a correlation to the number of autophagosomes. However, this measurement is deemed unreliable as LC3B II is also being degraded at the same time^44^. In our study, we found augmented levels of both proteins and can only speculate about the concomitant increase of LC3B I. In addition, we found a statistically significant increase of p62 levels for TBI and PF, but not for TBI + PF, compared to control (Fig. 6B; one-way ANOVA p = 0.0016). An increase in p62 is normally associated with impaired autophagic degradation, but since protein levels are affected both by decreased degradation as well as increased transcription/translation, we have investigated this further. Autophagosome degradation (autophagic flux) is measured by comparing protein levels in presence and absence of autophagy inhibitors^44,51^. One of these inhibitors is Baf A1, which prevents the fusion of autophagosomes with lysosomes and thus degradation^52–54^. Our results show that the levels of LC3B II and p62 are increased in the presence of Baf A1, since lysosomal degradation was hindered and the proteins accumulated (Fig. 6C), two-way ANOVA main column effect (experimental groups) p = 0.2756 (LC3B II) and p = 0.0038 (p62) and main row effect (pharmacological exposure) p = 0.0193 (LC3B II) and p < 0.001 (p62). These observed changes under Baf A1 signify functional autophagic degradation, as cells with a block in degradation would show no additional increase in the presence of the inhibitor. Furthermore, since the increase of p62 in the presence of the inhibitor is greater in the TBI and PF groups compared to control (+ Baf A1), but not for TBI + PF, the data suggests an upregulation of autophagy in those two groups.

The results obtained in the presence and absence of the autophagic inhibitor Baf A1 can further determine the amount of autophagosomes that were formed and would have been degraded in the observed time frame^55,56^. The mean values of the results shown in Fig. 6C are schematically represented and help to visualize how autophagosome synthesis and degradation can be calculated (Fig. 6D). Subtracting the values obtained at basal conditions Baf A1 (−) from the values in presence of Baf A1 (+) will determine the amount of autophagosomes that would have undergone degradation. Although not statistically significant, the amount of autophagosomes that would have been degraded shows the tendency to increase in the TBI and PF groups, but not in TBI + PF [Fig. 6E; one way ANOVA p = 0.8956 (LC3B II) and p = 0.2047 (p62)]. Correspondingly, this increase in the amount of autophagosomes could also be seen when calculating autophagosome synthesis by subtracting the value of the control group in the presence of Baf A1 from the value of a given experimental group in the presence of Baf A1. As before, this trend was observed in TBI cultures and cultures exposed to Aβ_42_ protofibrils, but not in cultures exposed to both, albeit not statistically significant [Fig. 6F; one way ANOVA p = 0.8661 (LC3B II) and p = 0.2999 (p62)].

These data allows the conclusion that autophagy is moderately upregulated in response to Aβ_42_ protofibrils exposure and TBI, whereas the combined exposure (TBI + PF) did not increase the autophagic activity of the cells.

### Immunocytochemistry confirms that the autophagy-lysosomal pathway is not impaired

**Figure 7:**
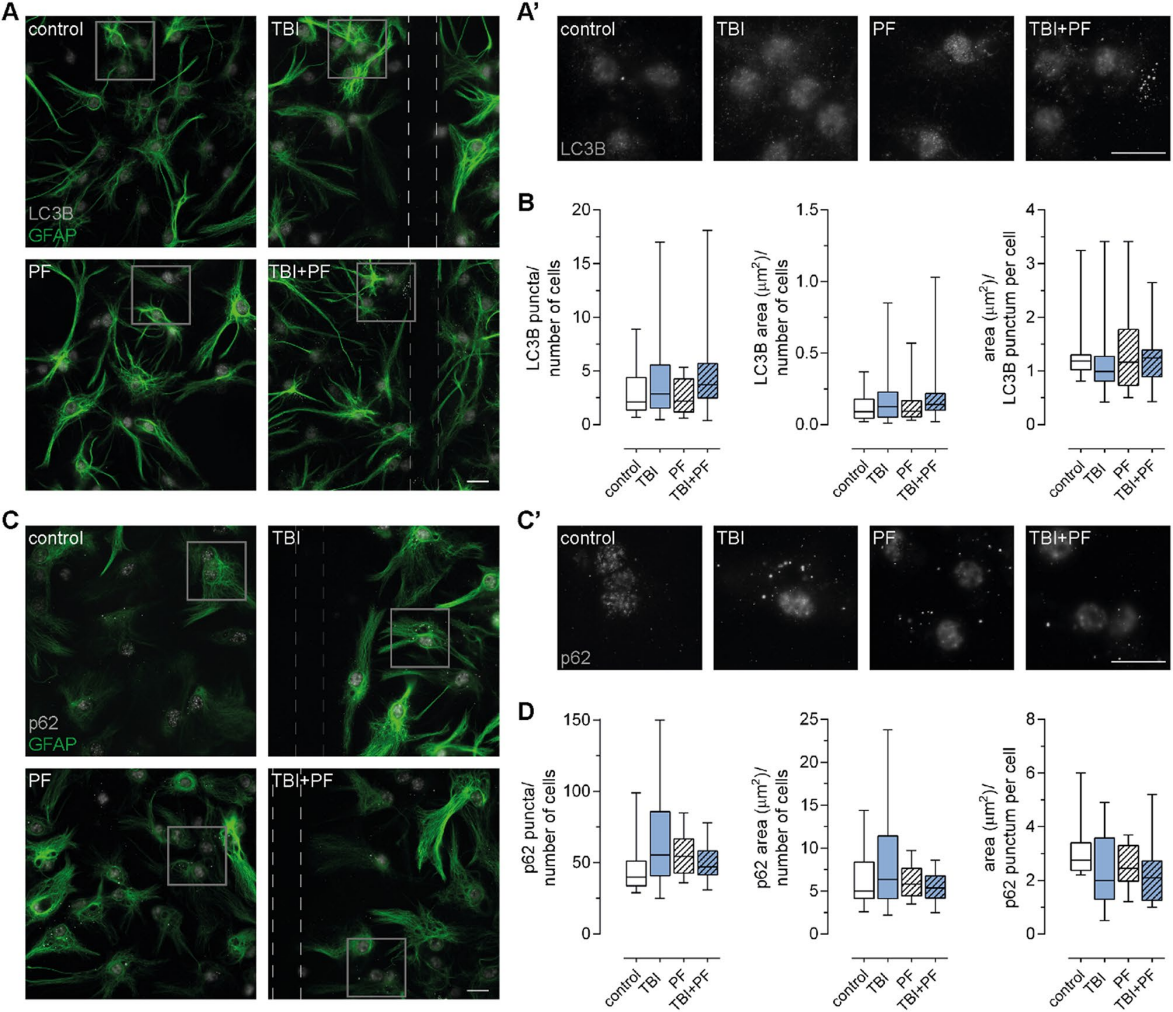
Immunocytochemistry confirms that the autophagy-lysosomal pathway is not impaired. (**A**) Immunostaining for LC3B and astrocytes (GFAP) were performed and representative fluorescence microscopy images are shown. (**B**) The number of LC3B puncta and their total area as well as the area per single LC3B-positive punctum were quantified. While the analysis did not reveal statistically significant differences, it was observed that some cells, in cultures that had undergone TBI alone or in combination with Aβ_42_ protofibril exposure, possessed higher amounts of LC3B puncta and subsequently also a greater total area measurement. (**C**) Representative images of cultures stained for p62 and astrocytes (GFAP) and (**D**) the quantification of p62 puncta, total area measurement and area per single p62-positive punctum. Again, cells in the TBI group occasionally possessed a much higher number of p62 puncta and total area measurement, albeit no statistically significant difference. The general tendency for an increase in p62 expression in the TBI and PF groups, observed in the immunoblotting data, was also identified in this image analysis. (**A′**,**C′)** Magnifications of the areas outlined in (**A**) or (**C**) are presented. Ten or five (LC3B and p62, respectively) images per independent cell culture (n = 2) per experimental group were analyzed and reported. Statistical analysis was performed using the Kruskal–Wallis test followed by Dunn’s multiple comparisons test, *p ≤ 0.05 **p ≤ 0.01 ***p ≤ 0.001. Scale bars 20 µm.

The use of imaging techniques to study alterations in the autophagy-lysosomal pathway have also been employed more recently, so we next thought to investigate LC3B and p62 expression by immunocytochemistry. Representative images of cultures stained for LC3B (Fig. 7A,A′) or p62 (Fig. 7C,C′) are shown, and LC3B- and p62-positive structures are visible as distinct puncta. We did not notice any changes to the localization of the puncta in exposed cultures compared to control. We then analyzed the amount of LC3B and p62 puncta quantitatively and also investigated if the area measurements were altered. While the analysis did not reveal statistically significant differences, it was observed that some cells possessed higher amounts of LC3B and p62 puncta and subsequently also greater total area measurements, especially in cultures that were subjected to TBI alone or in combination with Aβ_42_ protofibril exposure [Fig. 7B,D; Kruskal–Wallis test, puncta count: p = 0.0472 (LC3B) and p = 0.2173 (p62), total area: p = 0.1487 (LC3B) and p = 0.7411 (p62)]. Since impaired autophagy has been reported to result in autophagosomes of larger size^43,44,57^, we also calculated the area per LC3B- or p62-positive punctum. We did not find any statistically significant differences [Fig. 7B,D; Kruskal–Wallis test p = 0.2559 (LC3B) and p = 0.1777 (p62)]. However, in the case of p62, the puncta sizes actually showed a tendency to decrease in both experimental groups that were subjected to injury. Taken together, this data indicates that the autophagy-lysosomal pathway is not impaired. Differences to results obtained in western blotting could be explained by the fact that the two assays might measure different forms (soluble and detergent-insoluble) of the proteins^43,58^. The p62 puncta visualized with immunocytochemistry (ICC) are probably more representable of p62 inclusions/aggregates (in contrast to a diffuse cytoplasmic signal of soluble p62), whereas the western blotting in this study assessed detergent-soluble p62.

### Autophagic and endocytic compartments depicted by transmission electron microscopy

**Figure 8:**
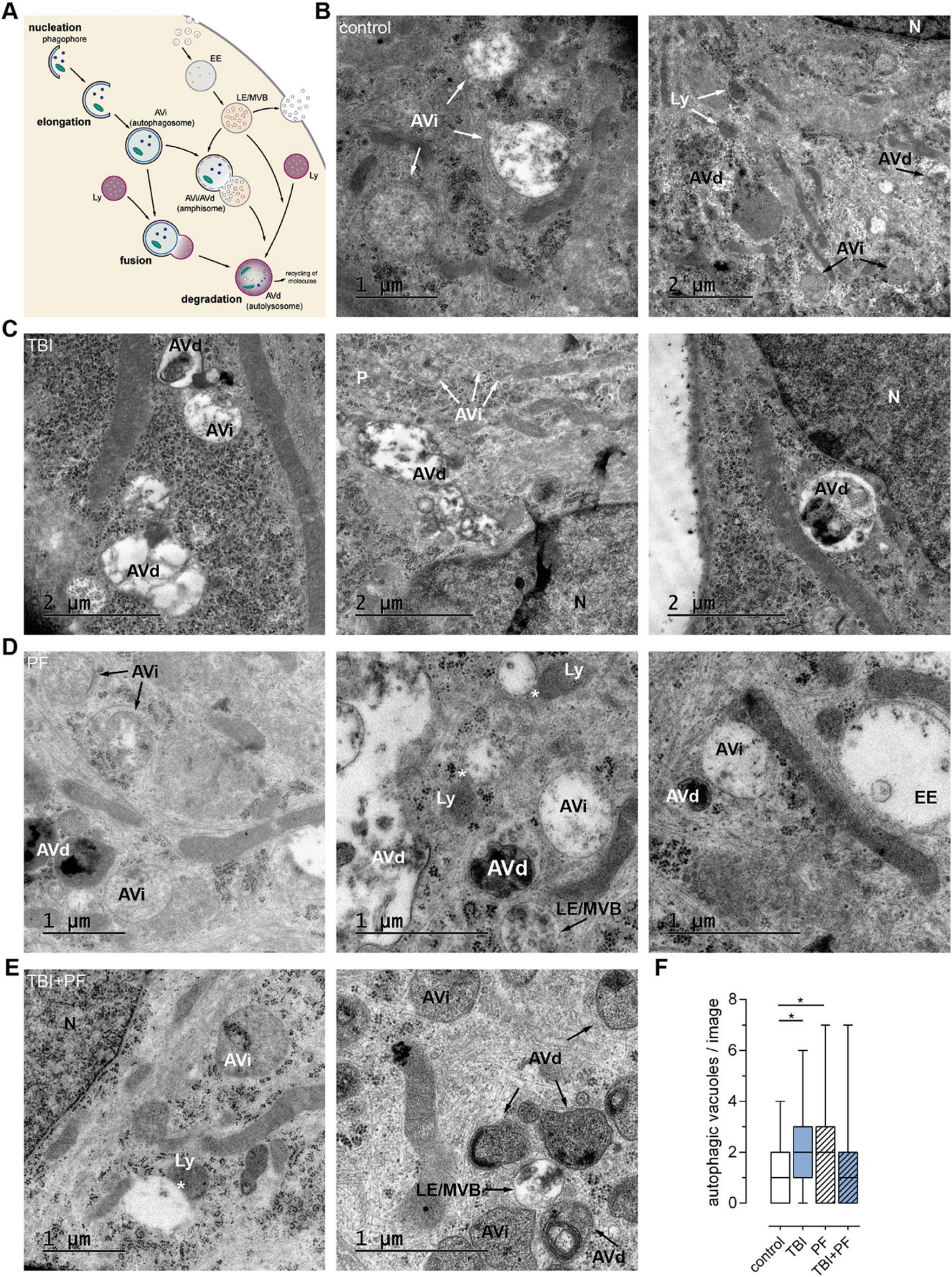
Autophagic and endocytic compartments depicted by transmission electron microscopy. (**A**) Schematic depiction of the autophagic and endocytic pathways used by the cells for the degradation and recycling of material. (**B**–**E**) TEM images of control (**B**), TBI (**C**), PF (**D**) and TBI + PF (**E**) cells. Autophagic and endocytic vacuoles at various stages of maturation are denoted. (**F**) The number of autophagic vacuoles (AVi and AVd) per TEM image was quantified; analyzing a total of 55–68 TEM images of five cells per experimental group. Statistical analysis was conducted using the Kruskal–Wallis test followed by Dunn’s multiple comparisons test, *p ≤ 0.05 **p ≤ 0.01 ***p ≤ 0.001. Potential fusion events are marked by stars (*). Abbreviations used: *AVd* degradative autophagic vacuole/autophagic compartment, *AVi* immature autophagic vacuole/autophagosome, *EE* early endosome, *LE* late endosome, *Ly* Lysosome, *MVB* multivesicular body, *N* nucleus, *P* phagophore/isolation membrane.

In order to complement our analysis of autophagy, we assessed the presence of autophagic vacuoles in control, TBI, PF and TBI + PF cells by TEM. It was actually the use of TEM that lead to the concept of autophagy being first described^59,60^ and the morphological analysis by TEM continues to be one of the most sensitive methods to study autophagic compartments. The autophagy-lysosomal pathway is depicted schematically in Fig. 8A and contains the following key steps: nucleation, elongation and cargo sequestration, followed by closure and maturation and finally fusion and degradation. The endo-lysosomal pathway is converging and therefore also displayed in the scheme (Fig. 8A). All these membrane-bound compartments contain a bulk of material with varying degrees of electron-density. Autophagosomes are characterised by their double limiting membranes. However, these might not always be decernable in conventional TEM, likely due to the extraction of lipids during sample preparation, and can thus not be used as the only identification criterion^61^. We have therefore also relied on their size, content and difference to more mature autophagic compartments. Maturing autophagosomes become more and more electron-dense as the cytoplasmic material they contain becomes partially degraded. TEM images of control (Fig. 8B), TBI (Fig. 8C), PF (Fig. 8D) and TBI + PF (Fig. 8E) cells show autophagic (and endocytic) vacuoles at various stages of maturation. Quantification confirmed that cells subjected to injury or exposed to Aβ_42_ protofibrils showed autophagic compartments (AVi and AVd) more often than control cells, but that there was no clear additative effect of the two (Fig. 8F; Kruskal–Wallis test p = 0.007).

## Discussion

Using a simplified in vitro model of Aβ pathology and TBI, we studied injury-induced processes in the presence of Aβ deposits in a neural-glial co-culture system. Our set-up was designed to mimic the situation in the aging brain and allow the investigation of cellular processes in response to physical brain trauma. TBI in the elderly population, known as geriatric TBI, presents a very common health problem as population-based studies have shown that the likelihood of falls and also the number of fall-related TBI cases increase with age^6,8^. This occurrence is accompanied by an increased probability and earlier onset of dementias in elderly TBI patients^9,10^. Although, not reflecting the full complexity of the brain pathology, in vitro trauma models allow for repeatable and controlled experiments, and agree well with in vivo TBI models^62–64^. Transection/scratch models, like ours, are well-established, simple to perform and produce precise injuries of reproducible levels. Moreover, it presents a very accessible system for further studies of primary and secondary injury processes. Following TBI, the clearance of cell debris is central in the attempt to preserve homeostasis and protect viable cells, and astrocytes are known to be a key player in this process^65^. Defective clearance of debris and deficiencies in other astrocytic functions have been observed in mice lacking GFAP and/or vimentin^66–69^. We have previously shown that the astrocytes in the neuro-glial co-culture model engulf dead cells and thereby save neurons from contact induced apoptosis^25^. Here, we also observed a stable number of neurons following experimental TBI. Likewise, there was no change in the number of neurons upon Aβ_42_ protofibril exposure. However, when combining Aβ exposure and TBI, the percentage of neurons in the culture decreased significantly. Previously, we have shown that Aβ-mediated neuronal death is caused by secondary mechanisms, specifically by the release of N-terminally truncated Aβ containing vesicles from astrocytes observed twelve days after the Aβ exposure pulse^17,18^. However, neuronal cell death in cultures exposed to both Aβ_42_ protofibrils and TBI was already observed after three days. There are several possible reasons behind this synergistic effect. Firstly, the release of internalized Aβ from injured astrocytes (due to the scratch) may be detrimental for the neurons. Another reason could be that the double burden of Aβ deposits and TBI impedes critical astrocytic functions and undermines their effort to maintain homeostasis, clear debris and protect neurons. The reaction of astrocytes could thereby determine the extent of damage. In response to brain trauma and other pathological conditions astrocytes become reactive, in a process termed astrogliosis, which involves long-lasting changes in astrocytic morphology and function^31,70–72^. Astrogliosis is also characterized by the upregulation of intermediate filament proteins, and by the secretion of inflammatory mediators and growth factors. GFAP upregulation under pathological conditions has readily been observed in vivo^32,65,73–76^. We assessed GFAP protein expression in our model system and observed wide heterogeneity between cells, which is also commonly observed in vivo^77^. Total GFAP fluorescence intensity measurements determined a statistically significant difference between cultures exposed to Aβ alone and cultures exposed to both Aβ and TBI. While higher GFAP levels were observed in the Aβ_42_ protofibril-exposed astrocytes, astrocytes subjected to TBI in the presence of Aβ deposits displayed lower GFAP levels. Although we have also previously seen that GFAP mRNA levels did not differ between injured and control cultures^25^, this was a surprising finding, since GFAP upregulation is regularly described following TBI and intermediate filament proteins are required for proper glial scar formation^67,69^. Another astrocytic protein studied in connection with TBI and other pathological conditions is the calcium-binding protein S100β, which is characteristically enhanced in the brain following trauma^33–35^. Our study revealed increased levels of S100β protein in astrocytes in all three experimental conditions investigated. This is also in line with reports that have found S100β levels to be elevated in tissue homogenates from AD patients and in astrocytes surrounding neuritic plaques in transgenic mouse models of AD^78,79^. Whether this upregulation of S100β in pathological conditions has beneficial or detrimental consequences is not fully determined. Notably, we observed a differential intracellular expression pattern of the two astrocytic markers GFAP and S100β in cells exposed to TBI in the presence of Aβ deposits. Varied expression patterns of astrocytic markers have also been observed in mouse models of AD and TBI. Differences in temporal and spatial expression of intermediate filament proteins in the context of Aβ pathology and injury in vivo has been suggested to depend on the stages of astrogliosis and inflammatory responses or subgroups of astrocytes^76,80^.

Interestingly, proteins like GFAP and S100β, may be released from astrocytes, especially during pathological conditions. It has been suggested that some of these secreted proteins may exert trophic or toxic effects in a concentration dependent manner—being neurotrophic at low levels, but causing neuronal death following augmented and prolonged release^34,35,81,82^. In this study we observed higher amounts of GFAP in the cell culture medium and in EV lysates in cell cultures subjected to TBI. Both proteins, GFAP and S100β, are increasingly explored as biomarkers, but how well they correlate with damage still needs to be fully established^65,81,83–85^.

There is collective evidence for mitochondrial dysfunction in neurodegeneration and in the context of other metabolic or environmental stresses^86–89^. The state of the mitochondrial network can thus serve as an indicator for disturbances to cellular homeostasis. Using CellLight Mitochondria-GFP labeling, we observed fragmented mitochondria and the leakage of mitochondrial proteins into the cytoplasm of TBI and/or Aβ_42_ protofibril-exposed astrocytes, which clearly speaks for a mitochondrial system in distress. Moreover, our TEM analysis displayed abnormal fusion events in those cells, which is another indicator for defective mitochondria. We have previously noted similar damage to the astrocytic mitochondrial network in Parkinson’s disease cell culture models^90–92^. Mitochondrial dysfunction is a prominent feature of Aβ-mediated pathology in neurons, in the AD brain and when studied in vitro^15,93–95^. Here, we could show that Aβ pathology and TBI can cause mitochondrial damage also in astrocytes. This might have detrimental consequences for key astrocytic functions and thereby exert knock-on effects for neurons and homeostasis in general.

In this study we have placed a special focus on autophagy. We have previously observed that astrocytes degrade engulfed material like cell corpses and aggregated proteins only gradually^15–18,25,91,92,96^. The presence of abnormal protein aggregates is a common feature of many neurodegenerative diseases and a failing autophagy system has been described as one of the possible causes^38,97–99^. This notion is supported by the fact that pharmacological activation of autophagy has been shown to reduce protein aggregates in model systems^100–103^. However, it may also be the other way around, so that the accumulation of protein aggregate results in an overwhelmed and dysfunctional autophagy system. Furthermore, there are also reports indicating that autophagic vacuoles are sites of Aβ production and that their incomplete degradation could contribute to excess Aβ levels^104,105^.

In this study, we noted an increased autophagic activity in cultures exposed to Aβ_42_ protofibrils and TBI alone. Interestingly, autophagy was not upregulated in cells subjected to TBI in the presence of Aβ deposits. Although not blocked, this failure to upregulate autophagy might be a sign for inefficient compensatory mechanisms when faced with the double burden of Aβ exposure and TBI, which could subsequently have detrimental effects. In earlier studies, the role of autophagy in neurodegenerative diseases has focused predominantly on neurons^22,99,106–108^. Our cell model system contains mainly astrocytes and thus our study gives new insights into the role of autophagy in glial cells. Given the essential role of astrocytes in brain homeostasis, the autophagy-lysosomal processing of protein aggregates and cellular debris has a central function in the influencing the trajectory of neurodegeneration.

## Conclusion

How geriatric TBI influences the development of dementia, and which cellular mechanisms are central to this link, is largely unknown. Employing an in vitro model, designed to mimic the conditions in the aging brain, we here demonstrated that the double burden of Aβ deposits and TBI results in increased acute death of neurons. It also causes altered astrocytic responses as measured by the changed expression of key astrocytic proteins, mitochondrial network disruptions and aberrant autophagic activity. Astrocytes are known to cope rather well with great amounts of Aβ deposits and are highly adaptive in response to injury. They will try to restore homeostasis whenever possible, but once astrocytes become dysfunctional it will have detrimental consequences. The link between trauma and the development of AD is probably complex, but this study contributes important findings and advances our understanding about underlying cellular processes and sheds new light on the regulation of autophagy in astrocytes.

## Materials and methods

### Materials

Reagents used in this study: Amyloid beta 1–42 (#SP-BA42, Innovagen and #H-8146, Bachem) and bafilomycin A1 (#196000, Merck). Antibodies were obtained from the following sources: mouse monoclonal βIII tubulin (clone TUJ1, #MMS-435P, Covance; 1:200), rabbit polyclonal GFAP [#Z0334, Agilent DAKO; 1:400 (ICC), 1:10,000 (WB) and 1 µg/ml (ELISA)] or mouse monoclonal GFAP [clone GA5, #MAB3402, Merck; 1:200 (ICC) or #G3893, Sigma-Aldrich; 5 µg/ml (ELISA)], mouse monoclonal CNPase (clone 11-5B, #C5922, Sigma-Aldrich; 1:500), mouse monoclonal Aβ (clone 6E10, #80300, BioLegend; 1:200), rabbit polyclonal LC3 [#51520, Abcam; 1:200 (ICC)], rabbit polyclonal LC3 [#NB100-2220, Novus Biologicals; 1:1000 (WB)], rabbit polyclonal p62 [#NBP1-48320, Novus Biologicals; 1:200 (ICC) and 1:1000 (WB)] and mouse monoclonal S100β [clone SH-B1, #S2532, Sigma-Aldrich; 1:200 (ICC) and 1:500 (WB)]. All secondary antibodies used for immunocytochemistry: anti-mouse or anti-rabbit Alexa Fluor conjugates (Invitrogen; 1:200). Secondary antibodies for western blotting: anti-mouse or anti-rabbit HRP-conjugates (Invitrogen; 1:20,000).

### Animals and ethical approval

The study is reported according to the ARRIVE guidelines. All animal experiments were approved by the Uppsala County Animal Ethics Board; ethical permit number: 5.8.18-08472/18. Housing and handling of the mice (C57BL/6) was performed in full compliance with rules and regulations of the Swedish Animal Welfare Agency and the European Communities Council Directive of 22 September 2010 (2010/63/EU). The mice were housed in a 12 h dark–light cycle, in an enriched environment and had access to food and water ad libitum.

### Cortical stem cell-derived cultures

Cultures were prepared as previously described^109^. Mouse embryonic cortices (gestation day 14) were dissected in Hank’s Buffered Salt Solution (HBSS, #14170, Gibco) supplemented with 8 mM Hepes buffer (#15630, Gibco), 100 U/ml Penicillin and 100 µg/ml Streptomycin (#15140, Gibco). The embryonic cortical stem cells were allowed to expand as neurospheres in serum-free proliferation medium containing Dulbecco's Modified Eagle Medium (DMEM/F12 with GlutaMAX, #31331, Gibco), 1 × B27 supplement (#17504, Gibco), 100 U/ml Penicillin, 100 µg/ml Streptomycin, 8 mM Hepes buffer and fortified with 10 ng/ml, basic fibroblast growth factor (bFGF, #13256029, Gibco) and 20 ng/ml epidermal growth factor (EGF, #354010, Corning) in non-treated tissue cultures flasks (at 37 °C, 5% CO_2_), with a passage every 2 to 3 days. For experiments, cells were seeded in a monolayer at a density of 3 × 10^4^ cells/cm^2^ on cover glasses (#630-2186, Marienfeld) coated with poly-l-ornithine (1:4 in dH_2_O, #P4957, Sigma-Aldrich) and laminin (1:1000 in phosphate-buffered saline (PBS), #23017015, Gibco). For the first 24 h cells were cultured (at 37 °C, 5% CO_2_) in proliferation medium (composition described above), thereafter in mitogen-free differentiation medium (DMEM/F12 with GlutaMAX, 1 × B27 supplement, 100 U/ml Penicillin, 100 µg/ml Streptomycin, 8 mM Hepes buffer), which was fully replaced every 2 to 3 days during the 7-day differentiation period. Being based on embryonic, cortical stem cells, this well characterized cell culture system solely contains cells of the neural lineage^25,110–112^. Differentiation led to a mixed population of 75 ± 8% astrocytes, 25 ± 8% neurons and 6 ± 3% oligodendrocytes, but not microglia, as expected from the literature^17,18,91^. Independent experiments were carried out using cells obtained from embryos of different pregnant mice.

### Synthetic Aβ_42_ protofibrils

Aβ_42_ protofibrils were prepared according to a well-established protocol^113,114^. Briefly, synthetic Aβ_42_ peptides were dissolved in 10 mM NaOH, diluted to 443 µM (2 mg/ml) with 10 × PBS and incubated for 30 min at 37 °C. Any insoluble aggregates were then removed by centrifugation at 17,900×*g* for 5 min. The Aβ_42_ protofibrils were diluted with PBS to a stock concentration of 100 µM and stored at − 70 °C. Analysis of the synthetic Aβ_42_ protofibril preparation was routinely performed by TEM (Supplementary Fig. 1), by using a protofibril specific ELISA and by size-exclusion chromatography^17,115,116^.

### Aβ_42_ protofibril exposure

Following a differentiation period of 7 days, the cell cultures were exposed to 0.1 µM Aβ_42_ protofibrils for 24 h (2 ml total volume/3.5 cm Ø well). Cell cultures of the control and TBI experimental groups received fresh medium without Aβ_42_ protofibrils. After the 24 h exposure pulse, the cells were rinsed twice to remove any excess Aβ, before being subjected to the scratch injury or cultured in Aβ-free differentiation medium (composition as above) for 3 additional days, upon which the culture medium was collected and the cells processed for immunocytochemistry, western blotting or TEM.

### In vitro injury

The mechanically injury was induced using a scalpel blade. Cell cultures were subjected to 20 cuts in a grid-like pattern: 10 vertical and 10 horizontal scalpel cuts, approximately 2 mm apart. Cells were then continued to be cultured in differentiation medium (composition as above) for 3 days, before being process for immunocytochemistry, western blotting or TEM. The cell culture medium was also collected at this point.

### Extracellular vesicle preparation

The conditioned cell culture medium of each experimental group was collected at the end of the experiment as described above. It was centrifuged at 300×*g* for 5 min, followed by another centrifugation at 2000×*g* for 10 min to remove free-floating cells and cell debris. Extracellular vesicles were then isolated from the medium by ultracentrifugation at 135,000×*g* for 1.5 h at 4 °C. The obtained pellets were re-suspended in PBS (for TEM analysis) or lysis buffer [for ELISA, see buffer composition below (Western blot analysis)].

### Autophagy regulation

To study the inhibition of autophagy, cells were incubated with the lysosomal inhibitor bafilomycin (500 nM) for 6 h before fixation.

### Immunocytochemistry

In experiments investigating mitochondria, the baculovirus-based CellLight Mitochondria-GFP reagent (#C10600, Invitrogen) was added to the cells 24 h before fixation according to manufacturer’s instruction.

Cells were fixed with 4% PFA/PBS for 15 min at room temperature (RT), followed by two washes in PBS. The cells were then permeabilized and blocked in 0.1% Triton X-100/PBS with 5% normal goat serum for 30 min at RT. Primary antibody incubations were performed for 1 h at RT and secondary antibody incubations for 30 min at 37 °C, both in 0.1% Triton X-100/PBS with 0.5% NGS. All incubations were followed by three washes in PBS for 5 min each. Cover glasses were mounted using EverBrite hardset mounting medium with DAPI (#23004, Biotium).

### Fluorescence microscopy and image analysis

Images were taken with a Zeiss Axio Observer Z1 wide-field microscope equipped with 20×/0.4 LD plan-neofluar, 40×/0.95 plan-apochromat and a 63×/1.40 oil DIC plan-apochromat objectives. For each staining, the image analysis was performed on at least five independent fields/cover glass per experimental group and independent cell culture (as stated in the figure legends).

The number of βIII tubulin-positive neurons in culture and the total cell number counts (identified by DAPI staining) were performed manually. Also, the mitochondrial network assessment was carried out manually. Mitochondrial health was defined according to well-characterized parameters^37^. The number of cells with fragmented mitochondria was normalized against the total number of CellLight Mitochondria-GFP transfected cells. Total fluorescent intensities (GFAP and S100β) were measured using macros developed in ImageJ or the Zeiss Zen software. LC3B and p62 signal was quantified using the Analyze Particle command in ImageJ. The number of LC3B and p62 puncta, the total area as well as the area/punctum was assessed and normalized to the number of living cells identified by DAPI nuclei staining.

### Western blot analysis

Cells were lysed in an appropriate amount of lysis buffer [20 mM Tris pH 7.5, 0.5% Triton-X-100, 0.5% Deoxycholic acid, 150 mM NaCl, 10 mM EDTA, 30 mM Na_4_O_7_P_2_ and supplemented with 1 × Halt Protease Inhibitor Cocktail (#78430, Thermo Scientific)]. Protein concentrations were determined using Pierce BCA Protein Assay kit according to manufacturer’s instruction (#23225, Thermo Scientific) and 18 µg of protein was prepared for SDS-PAGE by the addition of Bolt Sample Reducing Agent (#B0009, Invitrogen) and NuPAGE LDS Sample Buffer (#NP0007, Invitrogen). Samples were loaded on Bolt 4–12% Bis–Tris gels (Invitrogen) and electrophoresis performed at 200 V for 30 min, in 1 × MES SDS running buffer (#B0002, Invitrogen). Proteins were transferred to PVDF membranes (#LC2002, Invitrogen) using the Mini Blot Module (Invitrogen), in 1 × transfer buffer (Bolt Transfer Buffer (#BT00061, Invitrogen), 10% methanol, 0.1% Bolt Antioxidant (#BT0005, Invitrogen) and 0.01% SDS) at 20 V for 1 h. Membranes were blocked with 5% bovine serum albumin (BSA) in TBS with 0.1% Tween (TBS-T) for 1 h at RT. Primary incubations were performed at 4 °C overnight and secondary antibody incubations at RT for 1 h, both in 0.5% BSA in TBS-T. All incubations were followed by three washes in TBS-T for 10 min each. Proteins were visualized by chemiluminescence using Amersham ECL Prime Western Blotting Detection Reagent (RPN2232, GE) and imaged on the ChemiDoc XRS + (BIO-RAD). ImageJ or the ImageLab software (BIO-RAD) were used to quantify the intensities of the detected bands.

### Sandwich enzyme-linked immunosorbent assay

For GFAP detection in the cell culture medium and in EV lysates, high-binding 96 well polystyrene microplates (Corning) were coated with mouse monoclonal GFAP antibody (clone GA5, Sigma-Aldrich, 5 µg/ml in PBS pH 7.4) as the capture antibody at 4 °C overnight. Blocking of the plate was performed with 1% BSA in PBS pH 7.4 with 0.15% ProClin (all Sigma-Aldrich) for a minimum of 1.5 h at RT. Samples (medium samples 1:10, EV lysate samples 1:24) were diluted in ELISA buffer (0.1% BSA, 0.05% Tween 20, 0.15% ProClin in PBS pH 7.4, all Sigma-Aldrich) and incubated for 2 h at RT. The detection antibody rabbit polyclonal GFAP (DAKO, 1 µg/ml in ELISA buffer) was incubated for 1 h at RT, followed by an incubation with anti-rabbit IgG-HRP (#A0545, Sigma-Aldrich, 1:2000 in ELISA buffer) for 1 h at RT. K-Blue Aqueous TMB substrate (Neogen) was applied as HRP substrate and the reaction stopped with 1 M H_2_SO_4_ (Sigma-Aldrich). Washes were performed after every incubation (except between coat and block) with 0.1% Tween 20 and 0.15% ProClin in PBS pH 7.5 using a HydroSpeed micro-plate washer (TECAN). An Infinite M200 Pro microplate reader (Tecan) was used to measure the absorbance at 450 nm. ELISAs were repeated three times and the average reported.

### Transmission electron microscopy and image analysis

#### EVs

Purified EVs were mixed with an equal volume of 4% paraformaldehyde. A 5 µl drop of the sample was placed on a 200-mesh formvar/carbon coated grid. After 20 min the excess solution was removed by blotting on filter paper. The sample was washed in drops of PBS, 3 × 1 min, followed by 8 × 1 min washes in drops of Milli-Q H_2_O. The sample was stained in a drop of uranyl‐oxalate solution, pH 7.0 for 5 min, then stained in a drop of 4% uranyl acetate pH 4.0 + 2% methylcellulose (1 part uranyl acetate + 9 parts methylcellulose) on ice for 10 min. Excess of solution was removed by blotting on filter paper. Dried grids were visualized using a Tecnai G2 Spririt transmission electron microscope (FEI company) operated at 80 kV, equipped with an ORIUS SC200 CCD camera and Gatan Digital Micrograph software (both from Gatan Inc.).

#### Cells

Grown on Thermanox coverslips (#26031, Ted Pella), cells were fixed in 2.5% glutaraldehyde and 1% PFA in 0.1 M PIPES (pH 7.4). The cells were then rinsed with 0.1 M PIPES for 10 min and incubated in freshly made 1% osmium tetraoxide in 0.1 M PIPES for 30 min at RT. After incubation, the osmium tetraoxide was rinsed away with 0.1 M PIPES. Subsequently, the cells were dehydrated using increasing concentrations of ethanol (50%, 70%, 95% and Abs) for 10 min in each step. A layer of epoxy plastic (Agar 100 resin kit, #AGR1031, Agar Scientific) mixed with equal amounts of ethanol Abs, was added to the dishes and incubated for 1 h. The plastic/alcohol mix was poured off and new, pure, plastic was added onto the dishes for incubation overnight in a desiccator. The next day, the Thermanox coverslips were inverted onto blank BEEM capsules (Agar Scientific) and put in the oven for polymerization at 60 °C for 48 h. The Thermanox coverslips were detached by quickly placing them on a hot plate. Embedded cells were sectioned by using an EM UC7 Ultramicrotome (Leica). The sections were subsequently contrasted with 5% uranyl acetate and Reynold’s lead citrate and visualized with a Tecnai G2 Spirit transmission electron microscope (FEI company) operated at 80 kV, equipped with an ORIUS SC200 CCD camera and Gatan Digital Micrograph software (both from Gatan Inc.).

TEM images were analyzed to assess mitochondrial morphology and the presence of autophagic compartments. Five cells per experimental group were analyzed; quantifying in total between 55 and 68 images per group. Measurements of mitochondrial length and width were conducted in ImageJ and used to calculate the aspect ratio (mitochondrial length/width). Autophagic vacuoles (AVi and AVd) were quantified and reported as count/image.

### Statistical analysis

Results are presented as box plots (min to max), mean ± standard error of the mean (SEM) or mean with standard deviation (SD), as noted in the figure legends. Statistical analysis was performed in Prism 6 (GraphPad). Data were analyzed by one-way ANOVA or two-way ANOVA followed by Tukey's multiple comparisons tests. When the data did not follow Gaussian distribution for all groups, statistical analysis was performed using the Kruskal–Wallis test followed by Dunn’s multiple comparisons test.

## Supplementary Information

Supplementary Figures.

## Data Availability

Raw data are available from the corresponding author upon request.
