# Supplementary material for: Traumatic brain injury in the presence of Aβ pathology affects neuronal survival, glial activation and autophagy
Source: Sci Rep. 2021 Nov 26;11:22982. doi: 10.1038/s41598-021-02371-3 (PMC8626479; doi:10.1038/s41598-021-02371-3)
Supplement: Supplementary file 1 — Supplementary Figures. [file 41598_2021_2371_MOESM1_ESM.pdf]

Supplementary figures to:

Traumatic brain injury in the presence of Aβ pathology affects neuronal survival, glial activation and autophagy

Linn **Streubel-Gallasch**, Marlena **Zyśk**, Chiara **Beretta** and Anna **Erlandsson***

Department of Public Health and Caring Sciences, Uppsala University, Uppsala, Sweden.

* Correspondence to: Anna Erlandsson, Department of Public Health and Caring Sciences/ Molecular Geriatrics, Rudbeck Laboratory, Uppsala University, SE-751 85 Uppsala, Sweden. Phone number: +46 18 471 5060 E-mail: anna.erlandsson@pubcare.uu.se

Running title: TBI in the presence of Aβ pathology

Supplementary Figure 1 TEM characterization of Aβ_{42} protofibrils.

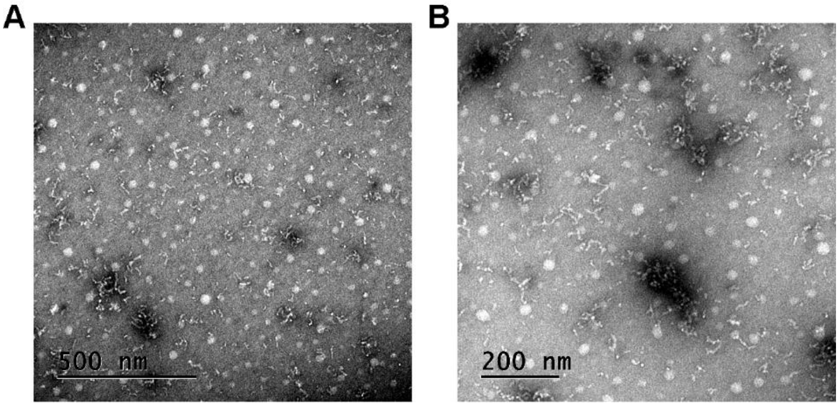

A and B) Aβ protofibril characterization using TEM. Scale bars 500 nm (A) and 200 nm (B). Negative staining of the Aβ protofibrils was performed using the following method: Aβ protofibrils (100 µM) were diluted 1:10 in Milli-Q H_2O and a 5 µl drop of the sample was placed on a formvar/carbon coated 200-mesh copper grid (Ted Pella). After 10 s, the excess solution was removed by blotting with filter paper. The sample was then directly stained with 2% uranyl acetate for 5 s. Excess of uranyl acetate was removed by blotting on filter paper. Dried grids were examined by TEM (Tecnai G2 Spirit) operated at 80kV.

Supplementary Figure 2 Western blot loading assessed by housekeeping gene GAPDH and total protein stain

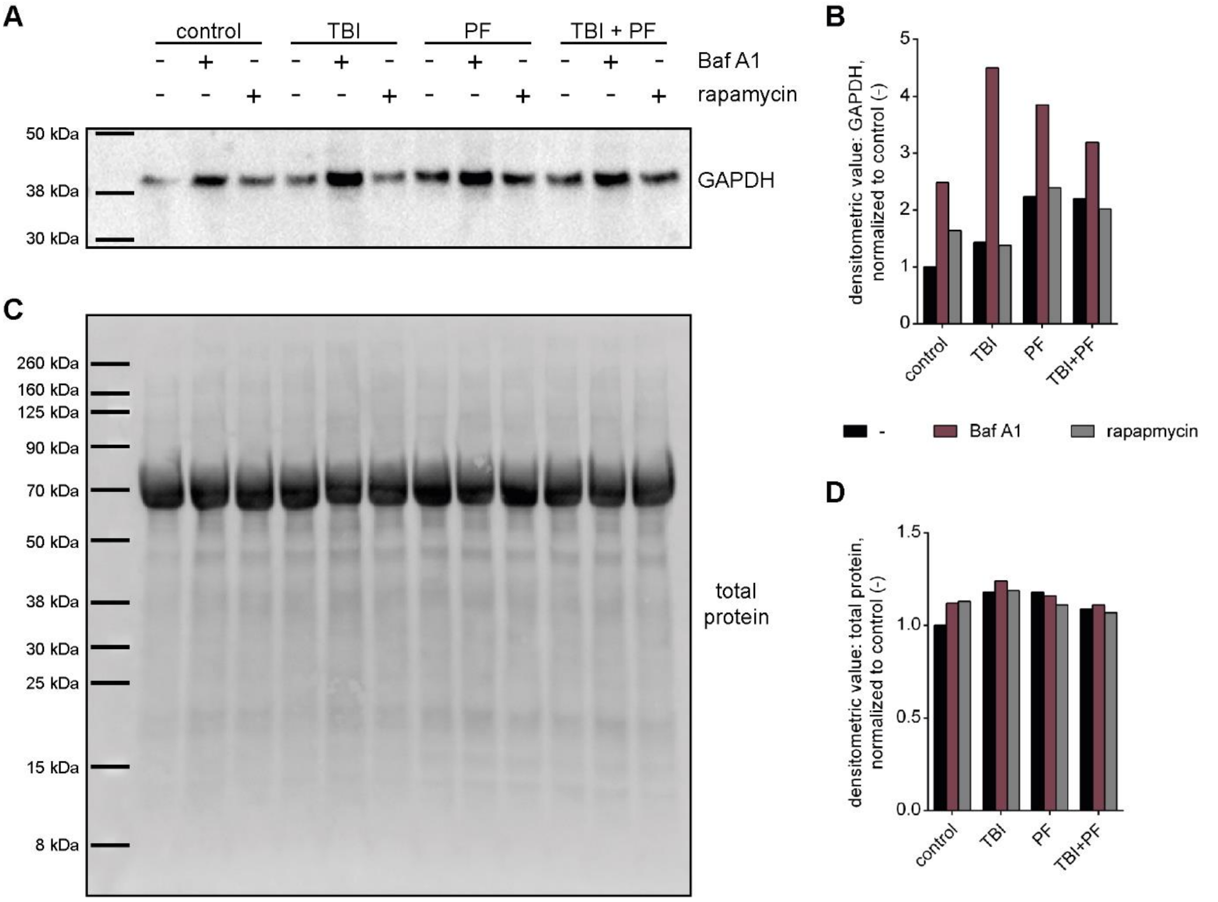

A) Representative blot (mouse monoclonal GAPDH, clone 1D4, #NB300-221, Novus Biologicals; 1:10000) and **B)** quantification of the detected GAPDH bands. Both, the experimental groups (TBI, PF and TBI+PF) and the use of the autophagy inhibitor Baf A1 (500 nM, 6 h) or inducer rapamycin (200 nM, 24 h), were found to influence the expression level of the housekeeping gene GAPDH and thus unsuitable as loading control and for protein normalization. **C)** Total protein staining of the membrane with No-Stain Protein Labeling Reagent (#A44717, Thermo Fisher) and **D)** its quantification confirmed equal loading when using protein concentrations determined by Pierce BCA Protein Assay kit.

Supplementary Figure 3 Full-length western blots

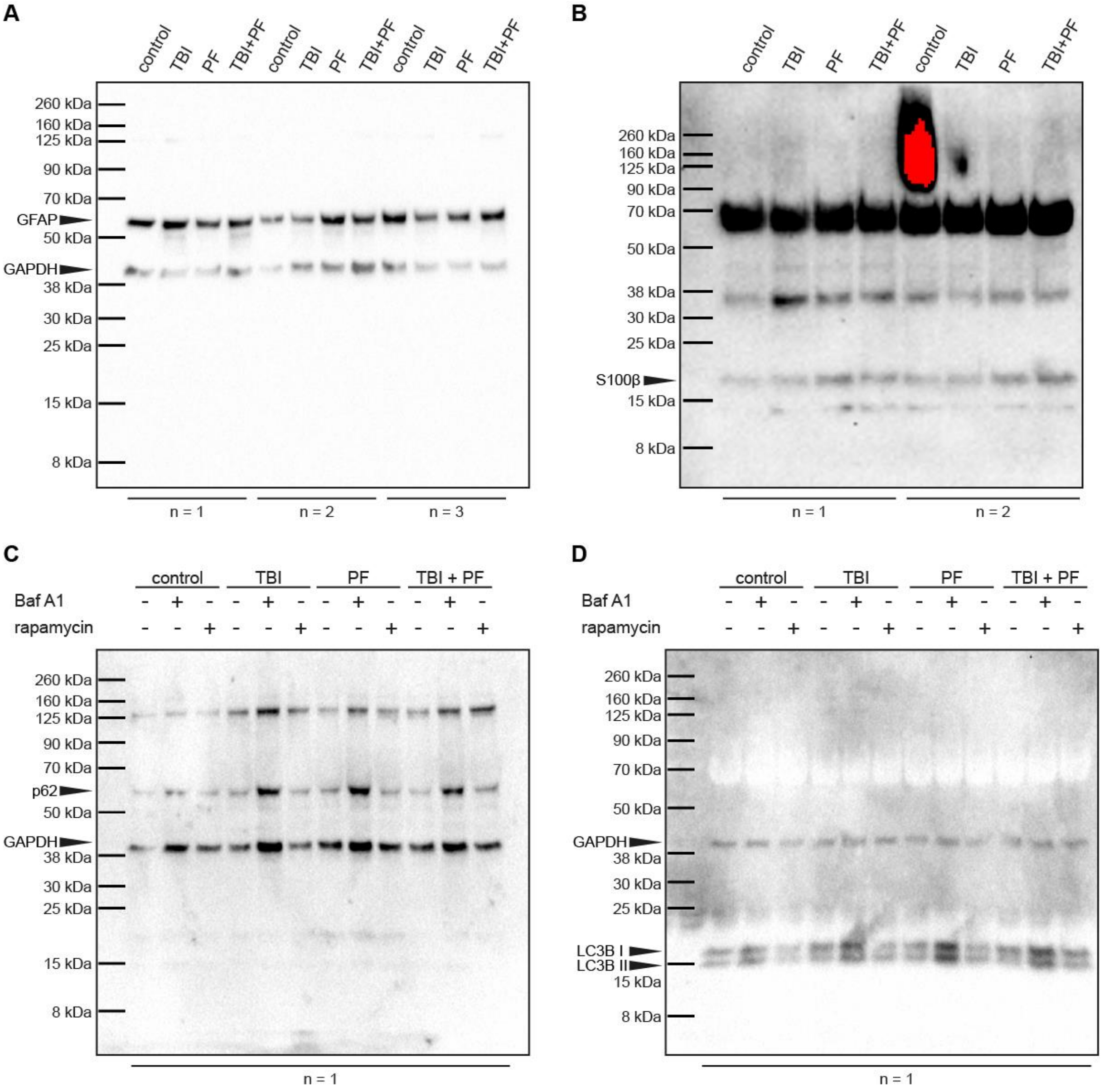

A) Full length blot of the cropped image shown in Figure 3C, GFAP rabbit polyclonal (#Z0334, Agilent DAKO; 1:10000). **B)** Full length blot of the cropped image shown in Figure 3G, S100b mouse monoclonal (clone SH-B1, #S2532, Sigma-Aldrich; 1:500). **C)** Full length blot of the cropped image shown in Figure 6A, p62 rabbit polyclonal (#NBP1-48320, Novus Biologicals; 1:1000). **D)** Full length blot of the cropped image shown in Figure 6A, LC3B rabbit polyclonal (#NB100-2220, Novus Biologicals; 1:1000). When indicated: GAPDH mouse monoclonal (clone 1D4, #NB300-221, Novus Biologicals; 1:10000).
